# The effects of the location of cancer stem cell marker CD133 on the prognosis of hepatocellular carcinoma patients

**DOI:** 10.1186/s12885-017-3460-9

**Published:** 2017-07-07

**Authors:** Yao-Li Chen, Ping-Yi Lin, Ying-Zi Ming, Wei-Chieh Huang, Rong-Fu Chen, Po-Ming Chen, Pei-Yi Chu

**Affiliations:** 1 School of Medicine, Kaohsiung Medical University, Kaohsiung, Taiwan; 2 Department of General Surgery, Changhua Christian Hospital, Changhua, Taiwan; 3 Transplantation Center, Third Xiangya Hospital of Central South University, Changsha, China; 4 Institute of Molecular and Genomic Medicine, National Health Research Institutes, 35 Keyan Road, Zhunan, Miaoli County,, 350 Taiwan Republic of China; 5 Research Assistant Center, Changhua Show Chwan Memorial Hospital, Changhua, Taiwan; 6 Department of Pathology, Show Chwan Memorial Hospital, No.542, Sec.1, Chung-Shang Road, Changhua City, Changhua County, 50008 Taiwan Republic of China; 7 School of Medicine, College of Medicine, Fu-Jen Catholic University, New Taipei City, Taiwan; 8 National Institute of Cancer Research, National Health Research Institutes, Tainan, Taiwan

**Keywords:** CD133, Prognosis, Hepatocellular carcinoma

## Abstract

**Background:**

CD133 (prominin-1) is widely believed to be a cancer stem cell marker in various solid tumor types, and CD133 has been correlated with tumor-initiating capacity. Recently, the nuclear location of CD133 expression in tumors has been discussed, but hepatocellular carcinoma (HCC) has not been included in these discussions. The goal of this study was to investigate the location of CD133 expression in HCC and this location’s potential value as a prognostic indicator of survival in patients with HCC.

**Methods:**

We enrolled 119 cancerous tissues and pair-matched adjacent normal liver tissue from HCC patients. These tissues were obtained immediately after operation, and tissue microarrays were subsequently constructed. The expression of CD133 was measured by immunohistochemistry (IHC), and the correlations between this expression and clinical characteristics and prognosis was estimated using statistical analysis.

**Results:**

The results showed that the CD133 protein expression levels of HCC in both the cytoplasm and nucleus were significantly higher than adjacent normal liver tissue. Kaplan–Meier survival and Cox regression analyses revealed that high CD133 expression in the cytoplasm was an independent predictor of poor prognosis for the overall survival (OS) and relapse-free survival (RFS) rates of HCC patients (*P* = 0.028 and *P* = 0.046, respectively). Surprisingly, high nuclear CD133 expression of HCC was an independent predictor of the good prognosis of the OS and RFS rates of HCC patients (*P* = 0.023 and *P* = 0.012, respectively).

**Conclusions:**

The clinical evidence that revealed cytoplasmic CD133 expression was correlated with poor prognosis, while nuclear CD133 expression was significantly correlated with favorable prognosis.

**Electronic supplementary material:**

The online version of this article (doi:10.1186/s12885-017-3460-9) contains supplementary material, which is available to authorized users.

## Background

Hepatocellular carcinoma (HCC) is the ninth most commonly diagnosed cancer in women, the fifth most commonly diagnosed cancer in men, and the second leading cause of cancer death worldwide, and HCC is most common in Asian and African populations [1, 2]. Hepatitis B virus (HBV), Hepatitis C virus (HCV), alcoholic liver disease, and nonalcoholic fatty liver disease have been identified as risk factors for HCC [3, 4]. The number of deaths that occur due to HCC is similar each year, which is a trend that highlights the aggressiveness of HCC [5]. Research has shown a hierarchy in which only a small subset of cells, including breast [6], colorectal cancer [7], glioblastoma [8], prostate cancer [9], and lung cancer [10] cells, drive cancer propagation and progression.

CD133 (also known as RP41, AC133, CD133, MCDR2, STGD4, CORD12, PROML1, and MSTP061) is a pentaspan transmembrane glycoprotein primarily identified in human hematopoietic stem and progenitor cells [11]. Recently, CD133 has widely been believed to be a potential marker of cancer stem cells, including HCC [12]. Importantly, CD133 can interact with p85 to activate PI3K/AKT/mTOR-signaling pathways in cancer stem cells, and this activation consequently provokes cancer stem cells to promote tumorigenic capacity [13].

Many studies have investigated whether CD133 expression is useful for clinical outcomes, and these studies have shown that CD133 is positively related to poor prognosis in HCC patients [14], that high CD133 levels are associated with shorter survival rates in rhabdomyosarcoma patients [15], and that CD133 expression might be an unfavorable prognosis for ovarian cancer patients [16]. Two meta-analyses have shown that higher CD133 levels are significantly associated with lymph node metastasis, clinical stage, and histopathological grade in colorectal cancer and esophageal carcinoma patients [17, 18].

Recently, a report of a triple-negative breast cancer case revealed the nuclear location of CD133 in a Caucasian woman with a histological diagnosis of high-grade invasive ductal breast carcinoma, as determined by immunohistochemistry [19]. CD133 has also been found in an exclusive nuclear location in rhabdomyosarcoma cell lines, with proportions of CD133 ranging from 3.4% to 7.5% [20]. However, the role of CD133 located in the nucleus of HCC remains largely unknown.

In this study, we studied 119 tumor specimens and the paired adjacent normal tissue that had not been exposed to chemotherapy or targeted therapy drugs before surgery, and we examined CD133 expression levels and location using immunohistochemistry. We further used Kaplan–Meier and Cox regression analysis to investigate whether the expression levels and location of CD133 and clinicopathologic parameters can be of independent prognostic value in HCC cases.

## Methods

### Patients

Primary tumor tissues were obtained from 119 HCC patients receiving surgical resection in Changhua Christian Hospital from July 2011 to November 2013. The initial characteristics and clinical outcomes were collected until death, censorship or loss of follow-up. For each patient, representative tissue cores of the HCC tumor parts were carefully collected and made into tissue microarray. This study was approved by the ethics committee of the Institutional Review Board of Changhua Christian Hospital. Informed consents were agreed from 119 HCC patients in accordance with the Declaration of Helsinki and were obtained at the time of their donation. The age of all patients was between 31 and 82 years (mean ± SD 63.7 ± 10.2). Clinical parameters and overall survival data were collected from chart review. The survival time was defined to be the period of time from the date of primary surgery to the date of death. The median follow-up time after surgery was 982 days and the median overall survival of all patients was 1092 days. During this survey, 39 patients died. On the basis of the follow-up data, 15 patients relapsed.

### Immunohistochemistry and scoring

Immunohistochemistry (IHC) was used to detect CD133 protein expression. The CD133 antibody (orb18124) was purchased from Biorbyt (USA). Paraffin-embedded HCC tissue sections (4-μm) on poly-1-lysine-coated slides were deparaffinized and rinsed with 10 mM Tris-HCl (pH 7.4) and 150 mM sodium chloride. Peroxidase was quenched with methanol and 3% hydrogen peroxide. Slides were then placed in 10 mM citrate buffer (pH 6.0) at 100 °C for 20 min in a pressurized heating chamber. After incubation with 1: 200 dilution of CD133 antibody (orb18124) for 1 h at room temperature, slides were thoroughly washed three times with phosphate-buffered salinen (PBS). Bound antibodies were detected using the EnVision Detection Systems Peroxidase/DAB, Rabbit/Mouse kit (Dako, Glostrup, Denmark). The slides were then counterstained with hematoxylin. At last, the slides were photographed with the microscope (BX50, OLYMPUS, Japan). Negative controls were obtained by performing all of the IHC steps, but leaving out the primary antibody. The immunohistochemical staining scores were defined as described previously [21] and the intensities of signals were evaluated by a board certified pathologist. The immunostaining scores criteria was defined as the cell staining intensity (0 = nil; 1 = weak; 2 = moderate; and 3 = strong) multiplied by the percentage of stained cells (0–100%), resulting in scores from 0 to 300. A score higher than mean score were defined as ‘high’ immunostaining, while a score equal to or lower than mean score was categorized as ‘low’ in tumor. Although CD133 is known to show both cytoplasmic and membranous staining, our results revealed that highly nuclear CD133 was observed using immunohistochemistry. Please also have a look at http://www.proteinatlas.org/ENSG00000007062-PROM1/cancer/tissue/liver+cancer#img?utm_source=custserv&utm_medium=email&utm_campaign=CSE.

Of a hepatocellular carcinoma sample, and the CD133 antibody (orb18124, Biorbyt) is used to recognize an epitope corresponding to residues NHQVRTRIKRSRKLADSNFKD (Additional file 1: Figure S1).

#### Cell lines

The liver cancer cell lines HepG2 and PLC-5 were obtained from the National Health Research Institutes (Taiwan) and cultured in Dulbecco’s modified Eagle’s medium (DMEM; Life Technologies) containing 0.1 mM sodium pyruvate, 10% FBS, 2 mM l-glutamine, 100 IU/mL penicillin, and 100 μg/mL streptomycin. Briefly, 5 × 10^5^ cells were respectively transfected with 10 μg of the lentiviral vector pLKO (control) or pLKO/shCD133 (target sequence GCGTCTTCCTATTCAGGATAT) which were purchased from the National RNAi Core Facility at Academic Sinica, Taiwan. After 48 h, CD133 expression was confirmed by CD133 antibody (orb18124) for Western blotting and β-actin was used as a loading control.

#### Western blotting

After whole cell protein extracts were prepared in ice-cold RIPA lysis buffer and quantified by BCA (bicinchoninic acid) protein assay, equivalent amounts of cell lysates were separated by 8–12% SDS polyacrylamide gel electrophoresis and transferred onto a polyvinylidene difluoride (PVDF) membrane, which was then blocked in 5% non-fat milk in PBST (1X Phosphate Buffered Saline Tween-20) and probed overnight at 4 °C with the primary antibodies against human CD133 antibody (1: 1000, orb18124, Biorbyt) and β-actin (Sigma-Aldrich Corp., St. Louis, MO, USA). Anti-mouse or anti-rabbit IgG conjugated to horseradish peroxidase was used as the secondary antibody for detection using an enhanced chemiluminescence (ECL) western blot detection system (Millipore, Bedford, MA, USA), and band intensities were quantified by densitometry (Digital Protein DNA Imagineware, Huntington Station, NY).

#### Immunofluorescence

2.5 × 10^4^ PLC-5/PLKO and PLC-5/shCD133 cells were respectively seeded on cover slips for 150 mins in complete medium and then fixed with 4% formaldehyde for 5 min at room temperature prior to immunofluoresence assay. Cells were washed with phosphate-buffered saline three times, treated with 0.1% Triton for 10 min, and blocked with 5% goat serum for 1 h, cells were then incubated with CD133 antibody (orb18124, Biorbyt) at 200X dilution at 4 °C overnight followed by binding with Alexa Flour 488 goat anti-Rabbit for green fluorescence by Leica DM2500 Upright Fluorescence Microscope.

### Statistical analysis

Paired-samples *t*-test and Chi-square analysis were conducted using SPSS software (Version 18.0 SPSS Inc., Chicago, IL, USA) for the relationship of clinical parameters with cytoplasmic and nuclear CD133 in hepatocellular carcinoma patients. Survival curves were plotted using the Kaplan–Meier method, survival data were analyzed using the log-rank test and variables related to survival were analyzed using Cox’s proportional hazards regression model for the influences of clinical characteristics and cytoplasmic and nuclear CD133 expression on OS and RFS in HCC patients. A value of P less than 0.05 was considered to be statistically significant.

## Results

### CD133 expression was found in the cytoplasm and nucleus in HCC

**Fig. 1:**
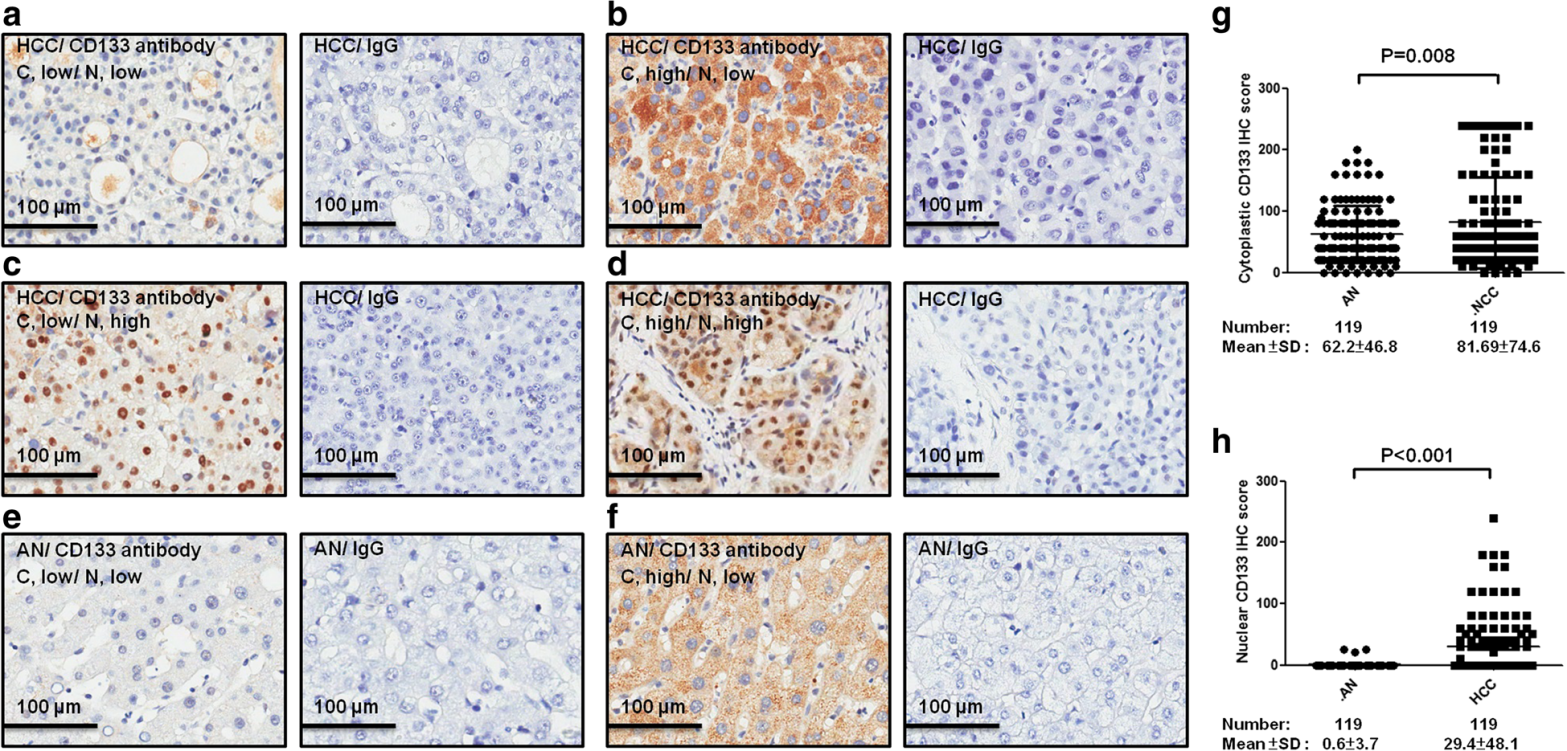
Immunohistochemistry showed the location of CD133 expression in the TU and AN of HCC patients. **a** A representative low C and low N CD133 immunostaining of HCC using the CD133 antibody (100 x). **b** A representative high C and low N CD133 immunostaining of HCC using the CD133 antibody (100 X). **c** A representative low C and high N CD133 immunostaining of HCC using the CD133 antibody (100 X). **d** A representative high C and high N CD133 immunostaining of HCC using the CD133 antibody (100 X). **e** A representative low C and low N CD133 immunostaining of AN using the CD133 antibody (100 X). **f** A representative high C and low N CD133 immunostaining of AN using the CD133 antibody (100 X). **g** The mean of the cytoplasmic CD133 scores was calculated in the TU and pair-matched AN, and the cytoplasmic CD133 scores were compared in the TU and pair-matched AN. **h** The mean of the nuclear CD133 scores was calculated in the TU and pair-matched AN, and the nuclear CD133 scores were compared in the TU and pair-matched AN. C: cytoplasm. N: nucleus. TU: tumor. AN: adjacent normal liver tissue. The corresponding isotype control of the CD133 antibody was obtained using normal rabbit IgG

A total of 119 HCC patients were enrolled in this study. CD133 expression was detected using immunohistochemistry in 119 hepatocellular tumors, and the representative results, which are shown in Fig. 1, show the cytoplasmic and nuclear locations of CD133. To investigate whether the cytoplasmic and nuclear locations of CD133 were linked with clinicopathological parameters, further statistical analysis was performed. The clinicopathological parameters that were studied, including age, gender, differentiation grade, tumor stage, hepatitis B surface antigen, and hepatitis C virus, were not significantly correlated with the cytoplasmic and nuclear locations of CD133 (see Table 1).

**Table 1:** Relationship of clinical parameters with cytoplasmic and nuclear CD133 in hepatocellular carcinoma patients

		CD133 (Cytoplasm)			CD133 (Nucleus)		
Variables	No.	Low	High	*p*	Low	High	*p*
Age (y/o)							
<65	64	44 (69)	20 (31)	0.485	36 (56)	28 (44)	0.413
≧65	55	41 (75)	14 (25)		35 (64)	20 (36)	
Gender							
Female	40	33 (83)	7 (17)	0.057	22 (55)	18 (45)	0.461
Male	79	52 (66).	27 (34)		49 (62)	30 (38)	
Differentiation							
Undifferentiation	4	3 (75)	1 (25)	0.551	2 (50)	2 (50)	0.703
Well	5	5 (100)	0 (0)		2 (40)	3 (60)	
Moderate	55	38 (69)	17 (31)		31 (56)	24 (44)	
Poor	53	37 (67)	16 (33)		35 (66)	18 (34)	
Stage							
I	42	32 (76)	10 (24)	0.429	24 (57)	18 (43)	0.657
II, III	75	52 (69)	23 (31)		46 (61)	29 (39)	
Hepatitis B surface antigen							
Negative	59	42 (71)	17 (29)	0.883	39 (66)	20 (34)	0.163
Positive	58	42 (72)	16 (28)		31 (53)	27 (47)	
Hepatitis C virus							
Negative	75	50 (67)	25 (33)	0.201	41 (55)	34 (45)	0.113
Positive	37	29 (78)	8 (22)		26 (70)	11 (30)	

*P* value was obtained from χ2 test

### Cytoplasmic and nuclear CD133 expression was higher in TU than in AN

CD133 expression was detected in different locations using IHC in 119 TU and the paired 119 AN tissues (Fig. 1a–f). The cytoplasmic CD133 expression level in HCC was significantly higher than the paired AN tissues (*P* = 0.008; see Fig. 1g), and nuclear CD133 expression was also significantly higher than the paired AN tissues (*P* < 0.001; see Fig. 1h). The mean scores of CD133 in the cytoplasmic and nuclear tumors were used for the cutoff values. A score greater than the mean was defined as high immunostaining, whereas a score equal to or less than the mean was categorized as low immunostaining.

### The validation of the CD133 antibody (orb18124)

**Fig. 2:**
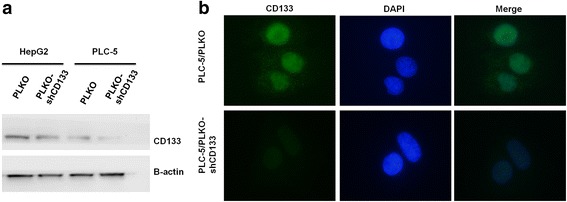
CD133 expression was decreased using the lentiviral vector pLKO/shCD133, and the CD133 antibody (orb18124, Biorbyt) was used to validate CD133 protein expression level and location in liver cancer cells. **a** CD133 expression was depleted upon transfection of HepG2 and PLC-5 cells with pLKO/shCD133. The CD133 protein expression levels were evaluated using western blotting. β-actin was used as a loading control. **b** CD133 antibody (orb18124, Biorbyt) was used to probe CD133 location in PLC-5 cells with pLKO and pLKO/shCD133 at 4 °C overnight, which was followed by binding the antibody with Alexa Flour 488 goat anti-Rabbit to produce green fluorescence, which was observed with a Leica DM2500 upright fluorescence microscope. The nuclei were stained with 4′,6′-diamidino-2-phenylindole (DAPI)

We used lentiviral vector pLKO (control) or pLKO/shCD133 (target sequence GCGTCTTCCTATTCAGGATAT), which were transfected into HepG2 and PLC-5 cells. Western blotting showed that the CD133 protein expression level decreased more in the HepG2 and PLC-5 cells that were transfected with pLKO/shCD133 than in the HepG2 and PLC-5 cells that were transfected with pLKO using the specific CD133 antibody (orb18124) (see Fig. 2a). We further examined the CD133 protein location in PLC-5/pLKO and PLC-5/pLKO/shCD133 with a Leica DM2500 upright fluorescence microscope by labeling CD133 antibody (orb18124, Biorbyt) with Alexa Flour 488 goat anti-Rabbit to produce green fluorescence in the antibody. The fluorescence images revealed that the cytoplasmic and nuclear CD133 protein expression was higher in the PLC-5/pLKO cells than in the PLC-5/pLKO/shCD133 cells. (see Fig. 2b).

### Different effects of OS and RFS on CD133 location of HCC

**Fig. 3:**
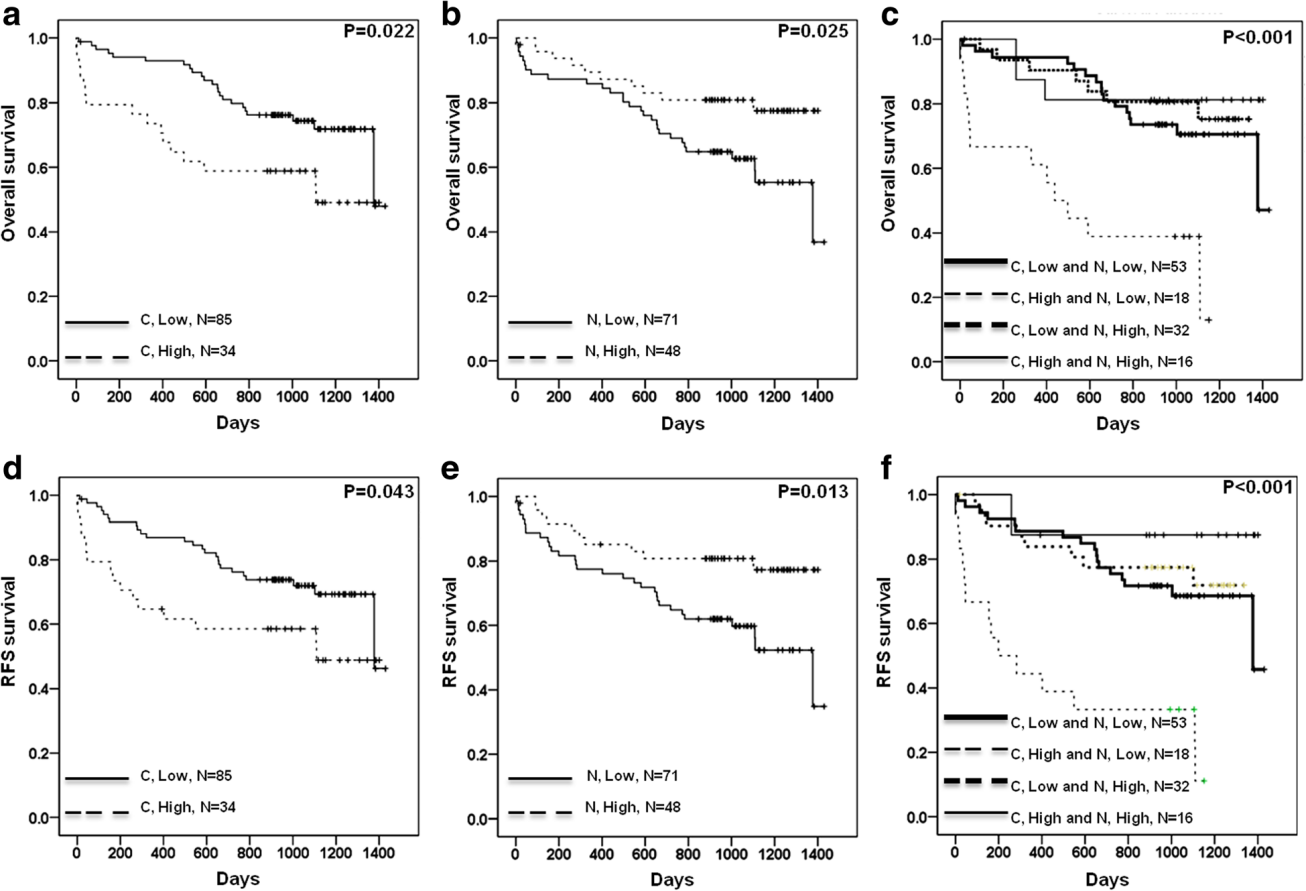
Kaplan–Meier plots of the OS and RFS rates in HCC patients based on cytoplasmic and nuclear CD133 expression levels. **a** The expression of cytoplasmic CD133 protein was examined on OS. **b** The expression of nuclear CD133 protein was examined on OS. **c** The expression of cytoplasmic and nuclear CD133 protein was examined on OS. **d** The expression of cytoplasmic CD133 protein was examined on RFS. **e** The expression of nuclear CD133 protein was examined on RFS. **f** The expression of cytoplasmic and nuclear CD133 protein was examined on RFS

We also investigated the association between clinicopathological parameters and CD133 with patient survival rates, and this association was statistically verified using univariate analysis. The results of this analysis showed that several characteristics, including age, gender, differentiation, tumor stage, hepatitis B surface antigen, hepatitis C virus, cytoplasmic CD133, and nuclear CD133, influenced the OS and RFS rates of HCC patients (OS: *P* = 0.330 for age, *P* = 0.761 for gender, *P* = 0.354 for differentiation, *P* = 0.003 for stage, *P* = 0.552 for hepatitis B surface, *P* = 0.152 for hepatitis C virus, *P* = 0.022 for cytoplasmic CD133, and *P* = 0.025 for nuclear CD133; RFS: *P* = 0.851 for age, *P* = 0.881 for gender, *P* = 0.179 for differentiation, *P* = 0.001 for stage, *P* = 0.861 for hepatitis B surface, *P* = 0.189 for hepatitis C virus, *P* = 0.022 for cytoplasmic CD133, and *P* = 0.013 for nuclear CD133; see Table 2). The Kaplan–Meier analysis showed that patients with a high level of cytoplasmic CD133 expression (C+) had shorter OS and RFS periods than patients with a low level of cytoplasmic CD133 (C-) expression (see Fig. 3a and d). Unexpectedly, we found that HCC patients with high nuclear CD133 expression (N+) had longer OS and RFS periods than patients with low levels of nuclear CD133 expression (N-) (see Fig. 3b and e).

**Table 2:** Univariate analysis of influences of clinical characteristics and cytoplasmic and nuclear CD133 expression on OS and RFS in hepatocellular carcinoma patients

		OS			RFS		
Characteristics	No.	Median survival (days)	Survival (%)	Log-rank	Median survival (days)	Survival (%)	Log-rank
Age (y/o)							
<65	64	1026	70.3%	0.330	999	67.2%	0.851
≧65	55	952	63.6%		952	63.6%	
Gender							
Female	40	1007	70.0%	0.761	1007	67.5%	0.881
Male	79	968	65.8%		954	64.6%	
Differentiation							
Moderate, Well	60	1047	70.0%	0.354	1026	70.0%	0.179
Poor, Undifferentiation	57	937	64.9%		1003	60.4%	
Stage							
I	42	1035	85.7%	0.003	1035	85.7%	0.001
II, III	75	934	57.3%		921	54.7%	
Hepatitis B surface antigen							
Negative	59	1003	69.5%	0.552	982	66.1%	0.861
Positive	58	953	63.8%		937	63.8%	
Hepatitis C virus							
Negative	75	934	62.7%	0.152	934	61.3%	0.189
Positive	37	994	75.7%		955	73.0%	
CD133 (Cytoplasm)							
Low	85	990	72.9%	0.022	990	72.9%	0.043
High	34	943	52.9%		944	52.9%	
CD133 (Nucleus)							
Low	71	946	59.2%	0.025	934	56.3%	0.013
High	48	1100	79.2%		1076	79.2%

We further stratified CD133 expression by dividing the study’s subjects into C−/N-, C+/N-, C−/N+, and C+/N+ groups to estimate the OS and RFS of HCC. The results showed that the C+/N- group had the shortest OS and RFS periods (see Fig. 3c and f). However, no statistically significant correlation was found between the C−/N-, C+/N-, C−/N+, and C+/N+ groups (C: cytoplasmic CD133; N: nuclear CD133) and age, gender, differentiation, tumor stage, HBV, and HCV. These results are shown in Additional file 2: Table S1.

### The location of CD133 is an independent prognostic index for HCC

Using Cox regression analysis, we found that CD133 location has prognostic significance for OS and RFS rates (see Table 3). The hazard ratios of C+ locations were 2.100 for OS and 1.946 for RFS when C- was used as a reference (95% CI = 1.082–4.075, *P* = 0.028 and 95% CI = 1.012–3.745, *P* = 0.046, respectively; see Table 3). However, the hazard ratios of N- locations were 2.347 for OS and 2.550 for RFS when N+ was used as a reference (95% CI = 1.122–4.907, *P* = 0.028 and 95% CI = 1.228–5.296, *P* = 0.012, respectively; see Table 3). In addition, the hazard ratios of stages II and III were 3.097 and 3.460 for OS and RFS when stage I was used as the reference (95% CI = 1.282–7.457, *P* = 0.012 and 95% CI = 1.441–8.308, *P* = 0.005, respectively; see Table 3). These results indicate that C+ and N- CD133 expression resulted in poor outcomes in HCC patients.

**Table 3:** Cox regression analysis for the influence of Stage and cytoplasmic and nuclear CD133 expression on OS and RFS in hepatocellular carcinoma patients

		OS				RFS		
Variables	HR	Unfavorable/Favorable	*p*	(95% CI)	HR	Unfavorable/Favorable	*p*	(95% CI)
CD133 (Cytoplasm)	2.100	High/ Low	0.028	1.082–4.075	1.946	High/ Low	0.046	1.012–3.745
CD133 (Nucleus)	2.347	Low/ High	0.023	1.122–4.907	2.550	Low/ High	0.012	1.228–5.296
Stage	3.092	II, III/ I	0.012	1.282–7.457	3.460	III, IV/ I, II	0.005	1.441–8.308

RR was adjusted for CD133 (Cytoplasm), CD133 (Nucleus) and tumor stage

## Discussion

The prognosis of HCC is mainly related to local invasion and intrahepatic metastasis, so the identification of novel methods that can effectively repress HCC malignancy is key for the management of HCC [22]. Interestingly, we noted higher nuclear CD133 expression in negative HCV-associated HCC. One previous study showed that chronic HCV infection appeared to predispose cells to gain cancer stem-like cell traits by upregulating CD133 expression [23], but nuclear CD133 has still not been reported in HCC.

In this study, we found not only cytoplasmic CD133 but also nuclear CD133 in HCC, and the expression of CD133 in the cytoplasm or nucleus of HCC was higher than pair-matched adjacent normal liver tissue (AN) (see Fig. 1). We also found that cytoplasmic CD133 expression was positively correlated with poor prognosis and that, inversely, nuclear CD133 expression was related to good prognosis (see Fig. 2 and Table 3).

According to the cancer stem cell (CSC) theory, CSCs are believed to represent only a minority of the tumor mass. CD133 has been applied as a marker for CSCs in several cancers [24–27]. Actually, CSCs are dependent on glycosylated CD133 protein, not native CD133 protein [28]. Recent studies have shown that high CD133 protein expression indicates a poor prognosis in various cancer patients [14–16, 29]. CD133 overexpression induces epithelial–mesenchymal transition (EMT) [30] and increases in vitro invasion and resistance to chemotherapy [31]. Interestingly, the Y828 phosphorylation level of CD133 can bind to P85 to activate PI3K/AKT pathways to promote tumorigenic capacity. In addition, CD133 transcription is upregulated by SP1 and Myc, and the inhibition of CD133 transcription is required for P53 tumor-suppressive activity and the methylated CpG islands of CD133 promoter [32].

Notably, another study showed that CD133 protein expression levels in both the cytoplasm and nucleus were significantly higher in non-small cell lung cancer (NSCLC) than in corresponding peritumoral tissue (these results agreed with our study), and high CD133 expression in both the cytoplasm and nucleus was associated with unfavorable outcomes in NSCLC [33]. Anomalous localization in the nucleus has been reported with several other cell-surface and secreted molecules in various cancers, and some molecules can move to the nucleus to be transcriptional factors, such as epidermal growth factor receptor, Cyr61-CTGF-NOV, epidermal growth factor, and fibroblast growth factor [34, 35]. Many endocytosed membrane proteins, including receptors for growth factors, cytokines, and hormones, are generally internalized by caveolin or clathrin-dependent endocytosis, which is delivered in the cytoplasm [36]. Therefore, we speculated that cytoplasmic CD133 could activate the signaling molecule. However, nuclear CD133 might play the role of rescue in highly expressed cytoplasmic CD133 during HCC progression, so the mechanism of nuclear CD133 in HCC should be further explored.

Collectively, our findings revealed that nuclear CD133 could confer good clinical outcomes in HCC patients regardless of cytoplasmic expression and that cytoplasmic CD133 was related to poor prognosis, which is a result that agreed with previous studies. Among these patients, the C+/N- group had the worst OS and RFS rates. Therefore, the blockage of cytoplasmic CD133 or the increase of nuclear CD133 is a beneficial strategy for targeted therapy.

## Conclusions

Our study revealed that HCC patients who highly expressed cytoplasmic CD133 had poorer clinical outcomes than those who lowly expressed cytoplasmic CD133. Conversely, HCC patients who highly expressed nuclear CD133 had better clinical outcomes than those who lowly expressed nuclear CD133. Collectively, the C+/N- group had the worst prognosis of all the studied groups.

## Additional files

Additional file 1: Figure S1. CD133 is known to show both cytoplasmic and membranous staining from the Human Protein Atlas of a hepatocellular carcinoma sample. (DOC 2903 kb)

Additional file 2: Table S1. Relationship of the clinical parameters with cytoplasmic and nuclear CD133 in hepatocellular carcinoma patients. (DOC 53 kb)

## Abbreviations

AN: Adjacent normal liver tissue
CSC: Cancer stem cell
EMT: Epithelial–mesenchymal transition
HBV: Hepatitis B virus
HCC: Hepatocellular carcinoma
HCV: Hepatitis C virus
IHC: Immunohistochemistry
OS: Overall survival
RFS: Relapse-free survival
TU: Tumor
