# Supplementary figures and images for: The effects of the location of cancer stem cell marker CD133 on the prognosis of hepatocellular carcinoma patients
Source: BMC Cancer. 2017 Jul 7;17:474. doi: 10.1186/s12885-017-3460-9 (PMC5501948; doi:10.1186/s12885-017-3460-9)

**Fig. S1**


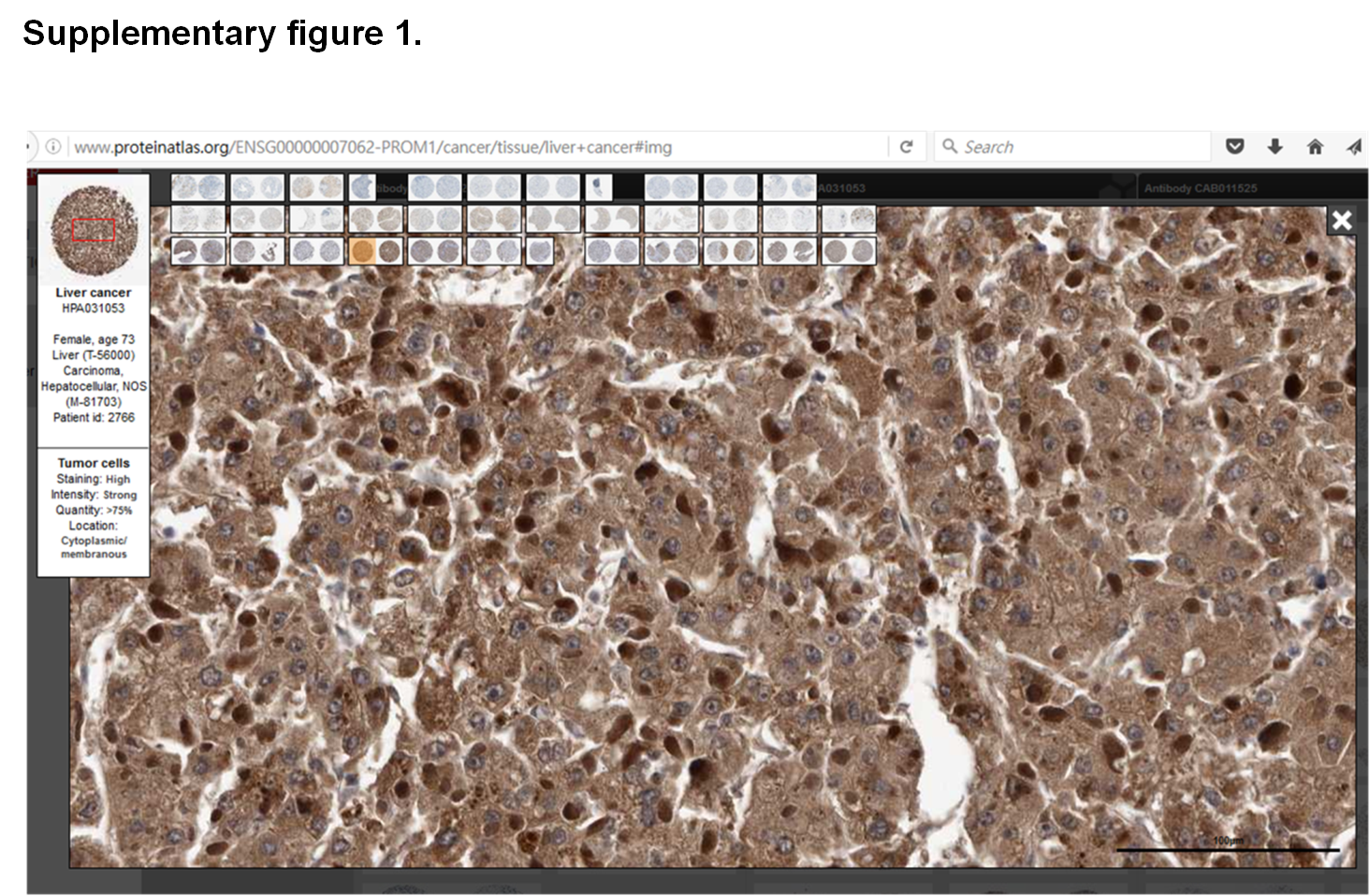

Supplement: Supplementary file 1 — CD133 is known to show both cytoplasmic and membranous staining from the Human Protein Atlas of a hepatocellular carcinoma sample. (DOC 2903 kb) [file 12885_2017_3460_MOESM1_ESM.doc]
