# Supplementary material for: The effects of the location of cancer stem cell marker CD133 on the prognosis of hepatocellular carcinoma patients
Source: BMC Cancer. 2017 Jul 7;17:474. doi: 10.1186/s12885-017-3460-9 (PMC5501948; doi:10.1186/s12885-017-3460-9)
Supplement: Supplementary file 2 — Relationship of the clinical parameters with cytoplasmic and nuclear CD133 in hepatocellular carcinoma patients. (DOC 53 kb) [file 12885_2017_3460_MOESM2_ESM.doc]

| Table S1. Relationship of clinical parameters with cytoplasmic and  nuclear CD133 in hepatocellular carcinoma patients. | | | | | | |
| --- | --- | --- | --- | --- | --- | --- |
|  |  | CD133 expression | | | |  |
| Variables | No. | C-/N- (%) | C+/N- (%) | C-/N+ (%) | C+/N+ (%) | p |
| Age (y/o) |  |  |  |  |  |  |
| ＜65 | 64 | 28 (45) | 8 (13) | 16 (25) | 12 (17) | 0.292 |
| ≧65 | 55 | 25 (45) | 10 (18) | 16 (29) | 4 (8) |  |
| Gender |  |  |  |  |  |  |
| Female | 40 | 18 (45) | 4 (10) | 15 (38) | 3 (7) | 0.161 |
| Male | 79 | 35 (44). | 14 (18) | 17 (22) | 13 (16) |  |
| Differentiation |  |  |  |  |  |  |
| Undifferentiation | 4 | 2 (50) | 0 (0) | 1 (25) | 1 (25) | 0.486 |
| Well | 5 | 2 (40) | 0 (0) | 3 (60) | 0 (0) |  |
| Moderate | 55 | 25 (45) | 6 (11) | 13 (24) | 11 (20) |  |
| Poor | 53 | 23 (43) | 12 (23) | 14 (26) | 4 (8) |  |
| Stage |  |  |  |  |  |  |
| I | 42 | 21 (50) | 3 (7) | 11 (26) | 7 (17) | 0.360 |
| II, III | 75 | 32 (43) | 14 (19) | 20 (27) | 9 (11) |  |
| Hepatitis B surface antigen |  |  |  |  |  |  |
| Negative | 59 | 29 (49) | 10 (17) | 13 (22) | 7 (12) | 0.552 |
| Positive | 58 | 23 (40) | 8 (14) | 19 (33) | 8 (13) |  |
| Hepatitis C virus |  |  |  |  |  |  |
| Negative | 75 | 28 (38) | 13 (17) | 22 (29) | 12 (16) | 0.255 |
| Positive | 37 | 21 (56) | 5 (14) | 8 (22) | 3 (8) |  |
| P value was obtained from χ2 test. | | | | | | |
| C: cytoplasmic CD133; N: nuclear CD133. | | | | | | |
| - : low; +: high. | | | | | | |
